# Performance evaluation of a newly developed three-dimensional model-based global-to-local registration in prostate cancer

**DOI:** 10.1093/jrr/rrz031

**Published:** 2019-05-28

**Authors:** Mitsuhiro Nakamura, Megumi Nakao, Hideaki Hirashima, Hiraku Iramina, Takashi Mizowaki

**Affiliations:** 1 Division of Medical Physics, Department of Information Technology and Medical Engineering, Human Health Sciences, Graduate School of Medicine, Kyoto University, Japan; 2 Department of Radiation Oncology and Image-Applied Therapy, Graduate School of Medicine, Kyoto University, Japan; 3 Department of Systems Science, Graduate School of Informatics, Kyoto University, Japan

**Keywords:** 3D model-based global-to-local registration, finite element method-based registration, Laplacian-based registration, Hausdorff distance, prostate cancer

## Abstract

We evaluated the performance of a newly developed three-dimensional (3D) model-based global-to-local registration of multiple organs, by comparing it with a 3D model-based global registration in the prostate region. This study included 220 prostate cancer patients who underwent intensity-modulated radiotherapy or volumetric-modulated arc therapy. Our registration proceeded sequentially, i.e. global registration including affine and piece-wise affine transformation followed by local registration. As a local registration, Laplacian-based and finite element method-based registration was implemented in Algorithm A and B, respectively. Algorithm C was for global registration alone. The template models for the prostate, seminal vesicles, rectum and bladder were constructed from the first 20 patients, and then three different registrations were performed on these organs for the remaining 200 patients, to assess registration accuracy. The 75th percentile Hausdorff distance was <1 mm in Algorithm A; it was >1 mm in Algorithm B, except for the prostate; and 3.9 mm for the prostate and >7.8 mm for other organs in Algorithm C. The median computation time to complete registration was <101, 30 and 16 s in Algorithms A, B and C, respectively. Analysis of variance revealed significant differences among Algorithms A–C in the Hausdorff distance and computation time. In addition, no significant difference was observed in the difference of Hausdorff distance between Algorithm A and B with Tukey’s multiple comparison test. The 3D model-based global-to-local registration, especially that implementing Laplacian-based registration, completed surface registration rapidly and provided sufficient registration accuracy in the prostate region.

## INTRODUCTION

The National Cancer Institute reported 164 690 new patients and 29 430 deaths from prostate cancer in 2018 in the USA [1]. For prostate cancer, 78.2% of patients are diagnosed at the early stage.

External-beam radiotherapy (EBRT) is one of the treatment approaches for early-stage prostate cancer. Although a meta-analysis showed that increasing the radiation dose significantly improves biochemical relapse-free survival [2], achieving this using conventional three-dimensional (3D) conformal radiation therapy is difficult due to the relationship between the mobility of the prostate and the larger margin size required to cover its movement, and the risk of toxicity to normal tissues around the prostate.

Among EBRT approaches, intensity-modulated radiation therapy (IMRT) and volumetric-modulated arc therapy (VMAT) are routinely used in clinical practice, which allow delivery of higher doses of radiation to the target while sparing surrounding normal tissues. However, IMRT is susceptible to geometric uncertainties due to steep dose gradients, resulting in lower dose delivery to the target and a higher dose delivered to the surrounding normal tissues. Therefore, accurate patient positioning is required to maximize the advantages of IMRT. As a supporting technique for achieving this, image-guided radiation therapy (IGRT) has been developed. When treating prostate cancer, daily cone-beam computed tomography (CBCT) images are typically acquired and co-registered to reference planning CT images using rigid registration software before beam delivery. Many investigators have reported that the shape of the rectum and bladder changes from day to day due to rectal gas and bladder filling, causing deviations in the target position in approved radiotherapy treatment plans [3–5]. During the image verification process, patient position is corrected only by translations and rotations, even when organs deform in a highly elastic way. Ideally, the daily dose is assessed at each fraction, and daily adaptive radiotherapy should be conducted for further improvement of the clinical outcomes; however, this deformation cannot be addressed with translation- and rotation-based registration.

Various 3D registration methods have been developed to address this issue [6]. 3D global registration, such as affine transformation and thin-plate splines (TPS) [7] without local registration, lead to large registration errors for regions of high curvature with spatially discontinuous changes in correspondence across sliding organ boundaries [8]. Meanwhile, local registration alone, such as B-spline and finite element methods (FEM), can be used to determine the correspondence point via maximum likelihood estimation, but this often results in erroneous registration of organs with large deformation [9].

To overcome the limitations of previous methods, we have developed a 3D model-based global-to-local registration of multiple organs. Our approach transforms a template model into a target model based on sequential registration, i.e. from global registration including affine and piece-wise affine transformation to local registration. As a local registration, FEM- [10, 11] and Laplacian-based registration [12–14] were implemented. FEM-based registration solved the problem of optimizing force constraints for the template model. The template model was deformed so as to represent the surface of the target model as closely as possible. Suwelack *et al.* applied FEM to match the intraoperative shape changes of the liver [11]. Meanwhile, the Laplacian-based registration was a least-squares problem for a template and a target model. An evaluation function on positional and discrete Laplacian constraints was defined. Kim *et al.* developed a statistical hippocampal model using the Laplacian-based registration [13]. Compared with the shape variations in these studies, organs in the prostate region often show larger shape differences among patients according to their physical and/or physiological condition. Investigation of the performance of the 3D model-based global-to-local registration of multiple organs will contribute to improvement of registration accuracy for prostate cancer.

We assumed that the accuracy of the 3D model-based global-to-local registration would depend on the number of patients included during construction of template models and on the registration algorithms employed; however, to date, there has been insufficient literature on this subject. First, we examined the impact of the number of patients on the accuracy of the 3D model-based global-to-local registration. Secondly, we assessed the accuracy of the 3D model-based global-to-local registration by evaluating its performance for 200 prostate cancer patients.

## MATERIALS AND METHODS

### Patients

The clinical data used in this study were from 220 randomly selected prostate cancer patients who underwent IMRT or VMAT in the prone position between July 2007 and September 2015. The median age was 72 years (range 49–84 years), and the clinical T-stage was T1c for 70 patients, T2a for 55 patients, T2b for 24 patients, T2c for 21 patients, T3a for 42 patients and T3b for 8 patients. The patients were numbered sequentially according to the start date of irradiation.

Written informed consent was obtained from the patients regarding the use of their clinical data for research and publication purposes. This study was performed in accordance with the Declaration of Helsinki and approved by our institutional review board (approval number R1446).

### CT simulation

Patients were immobilized in the prone position with a thermoplastic shell (Hip Fix system; CIVCO Medical Solutions, Kalona, IA, USA) that extended from the mid-thigh to the upper third of the leg, in combination with a vacuum pillow (Vac-Lok system; CIVCO Medical Solutions) and a leg support. Each patient underwent pretreatment planning CT scans (LightSpeed RT; GE Healthcare, Little Chalfont, UK) of 2.5 mm slice thickness. All patients were instructed to void the bladder and rectum ~1–1.5 h before the CT simulation, according to their individual urinary conditions.

The prostate, seminal vesicles (SVs), rectum and bladder were manually contoured by several experienced radiation oncologists and medical physicists. The rectum was determined as the area from 15 mm below the prostate apex to 15 mm above the tips of the SVs or prostate base. Details of our contouring protocol have been reported previously [15].

The contours of the prostate, SVs, rectum and bladder on planning CT images were converted to polygon (PLY) file format using a commercially available system [ITEM’s Viewer planning and Assistant System (iVAS); ITEM Corporation, Osaka, Japan].

### Flow of registration

**Fig. 1.**
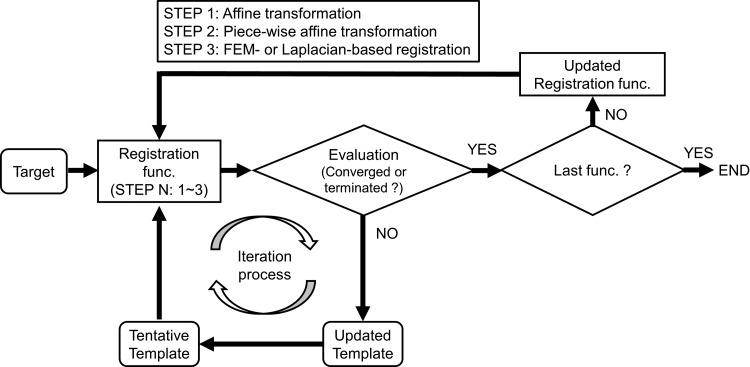
The schematic flow of registration. At STEP 1, the template models were updated until the convergence criterion or termination criterion was met. The convergence criterion was that the average mean bidirectional difference between after the end of the *k* – 1th loop and after the end of the *k*th loop in the most recent 10 iterations was <0.001 mm. Otherwise, the iteration process was terminated when the optimization calculation completed the 3000th iteration. When the convergence criterion or termination criterion was met, the iteration process for STEP 1 was terminated, and the iteration process for STEP 2 was then initiated. These processes were repeated until the convergence criterion or termination criterion was met in the last STEP. Once registration was completed for one target model, the iteration process was initiated for the next target model.

Figure 1 illustrates the schematic flow of registration. First, patient 1 was selected as the initial template model. Next, the corresponding template models with the same vertices and mesh topology were obtained by shape matching from *k* patients. As the template models have point-to-point correspondence, the average template model can be obtained by calculating the average of each coordinate. In this study, we used it as the template model. Registration was performed for each organ individually using one of the algorithms below.

- **Algorithm A:** sequential registration from global registration, including (STEP 1) affine and (STEP 2) piece-wise affine transformation, to (STEP 3) Laplacian-based registration was conducted [12].
- **Algorithm B:** sequential registration from global registration, including (STEP 1) affine and (STEP 2) piece-wise affine transformation, to (STEP 3) FEM-based registration was conducted [10].
- **Algorithm C:** only global registration, including (STEP 1) affine and (STEP 2) piece-wise affine transformation, was conducted.

During the iteration process, the template model for each organ was compared with a target model in PLY file format. The mean bidirectional distance between the template and the target model was employed as an objective function [16]. At STEP 1, the template models were updated until the convergence criterion or termination criterion was met. The convergence criterion was that the average mean bidirectional difference between after the end of the (*k* – 1)th loop and after the end of the *k*th loop in the most recent 10 iterations was <0.001 mm. Otherwise, the iteration process was terminated when the optimization calculation completed the 3000th iteration. When the convergence criterion or termination criterion was met, the iteration process for STEP 1 was terminated, and the iteration process for STEP 2 was then initiated. These processes were repeated until the convergence criterion or termination criterion was met in the last STEP. Once registration was completed for one target model, the iteration process was initiated for the next target model.

The algorithms were applied and implemented using in-house software written in C++.

### Evaluation

Two experiments were conducted in this study. In the first, the template models for the organs were constructed from the first 10, 20 and 50 patients, and 3D model-based global-to-local registration using Algorithms A and B was then performed on the prostate, SVs, rectum and bladder for 100 patients (numbers 51–150). In the second experiments, the template models for these organs were constructed from the first 20 patients, and then three different registrations, using Algorithms A, B and C, were performed on the organs for the remaining 200 patients (numbers 21–220).

The Hausdorff distance [17] was calculated for the surfaces of the organs in both experiments. The experiment was conducted on a personal computer with 32 GB RAM and an Intel Xeon E5-2687W v4 processor (dual-core CPU; 3.0 GHz).

The differences in Hausdorff distances and times to complete registration among Algorithms A–C were assessed by analysis of variance (ANOVA). Tukey’s multiple comparison test was also conducted to assess the difference of Hausdorff distance among Algorithms A–C for the remaining 200 patients. A *P*-value of 0.05 was considered statistically significant. All statistical analysis was performed using SPSS v.25.0 (IBM Corp., Armonk, NY, USA).

## RESULTS

### Volume characteristics

Table 1 summarizes the means ± standard deviation prostate, SVs, rectum and bladder volumes. In order of descending mean volume, the largest organ was the bladder, followed by the rectum, prostate and SVs.

**Table 1.** Mean ± standard deviation (range) volume of each organ

	No. of patients used to construct template models			No. of patients to be registered	
	10 pts.	20 pts.	50 pts.	100 pts.	200 pts.
Prostate (cm^3^)	30.9 ± 16.6	32.5 ± 16.1	28.4 ± 12.3	26.0 ± 8.9	26.8 ± 10.6
	(17.4–65.2)	(12.1–65.2)	(12.1–65.2)	(9.0–76.6)	(9.0–90.5)
Seminal vesicles (cm^3^)	4.1 ± 1.9	4.9 ± 2.5	6.4 ± 3.4	6.7 ± 3.6	7.0 ± 3.5
	(1.7–7.3)	(1.7–10.5)	(1.7–19.8)	(1.3–19.4)	(1.3–19.8)
Rectum (cm^3^)	50.9 ± 9.8	59.4 ± 24.4	64.9 ± 33.9	56.0 ± 19.5	59.9 ± 25.2
	(38.5–69.6)	(38.5–124.2)	(29.9–203.6)	(23.6–121.4)	(23.6–203.6)
Bladder (cm^3^)	80.6 ± 27.6	117.8 ± 66.1	127.5 ± 68.8	156.8 ± 83.9	150.0 ± 79.9
	(42.0–121.5)	(42.0–288.0)	(42.0–316.7)	(56.7–427.6)	(55.1–442.6)

Abbreviation: pts. = patients.

### Impact of the number of patients used to construct the template models on registration accuracy

Table 2 summarizes the Hausdorff distance using Algorithms A and B. The 75th percentile Hausdorff distance was <0.8 mm for all organs with Algorithm A; no significant difference was observed among the organs.

**Table 2.** Median Hausdorff distance with Algorithms A and B by the number of patients used to construct the template models

	10 pts.	20 pts.	50 pts.	*P*-value
Algorithm A				
Prostate (mm)	0.23 (0.20–0.29)	0.24 (0.20–0.30)	0.23 (0.19–0.27)	0.61
Seminal vesicles (mm)	0.37 (0.29–0.47)	0.38 (0.28–0.51)	0.35 (0.28–0.47)	0.84
Rectum (mm)	0.53 (0.42–0.69)	0.52 (0.42–0.62)	0.62 (0.49–0.76)	0.87
Bladder (mm)	0.40 (0.33–0.50)	0.41 (0.32–0.52)	0.40 (0.34–0.47)	0.57
Algorithm B				
Prostate (mm)	0.28 (0.23–0.34)	0.26 (0.23–0.34)	0.26 (0.23–0.32)	0.99
Seminal vesicles (mm)	0.85 (0.54–1.33)	0.69 (0.45–1.07)	0.64 (0.48–1.31)	0.27
Rectum (mm)	0.95 (0.66–1.44)	0.90 (0.68–1.40)	0.93 (0.70–1.31)	1.00
Bladder (mm)	0.66 (0.46–1.36)	0.59 (0.43–1.00)	0.53 (0.41–0.87)	<0.05

The number of patients used for validation of registration accuracy was 100. The interquartile range is shown in parentheses.

Algorithm A = 3D model-based global-to-local registration (Laplacian-based registration was used as a local deformable registration); Algorithm B = 3D model-based global-to-local registration (finite element method-based registration was used as a local deformable registration).

Abbreviation: pts. = patients.

With Algorithm B, the 75th percentile Hausdorff distances were >1 mm for the SVs and rectum and <0.4 mm for the prostate. No significant difference was observed among the organs. The Hausdorff distances became significantly smaller when larger numbers of patients were used to construct the bladder template models.

When 10 patients were used to construct template models, the ratios of the median Hausdorff distance with Algorithm B to that with Algorithm A were 1.2, 2.3, 1.8 and 1.7 for the prostate, SVs, rectum and bladder, respectively. With increasing numbers of patients used to construct the template models, the ratios became smaller, but were >1 for all organs (1.1, 1.8, 1.5 and 1.3 for the prostate, SVs, rectum and bladder, respectively).

### Comparison of registration accuracy between 3D model-based global-to-local registration and 3D model-based global registration

**Fig. 2.**
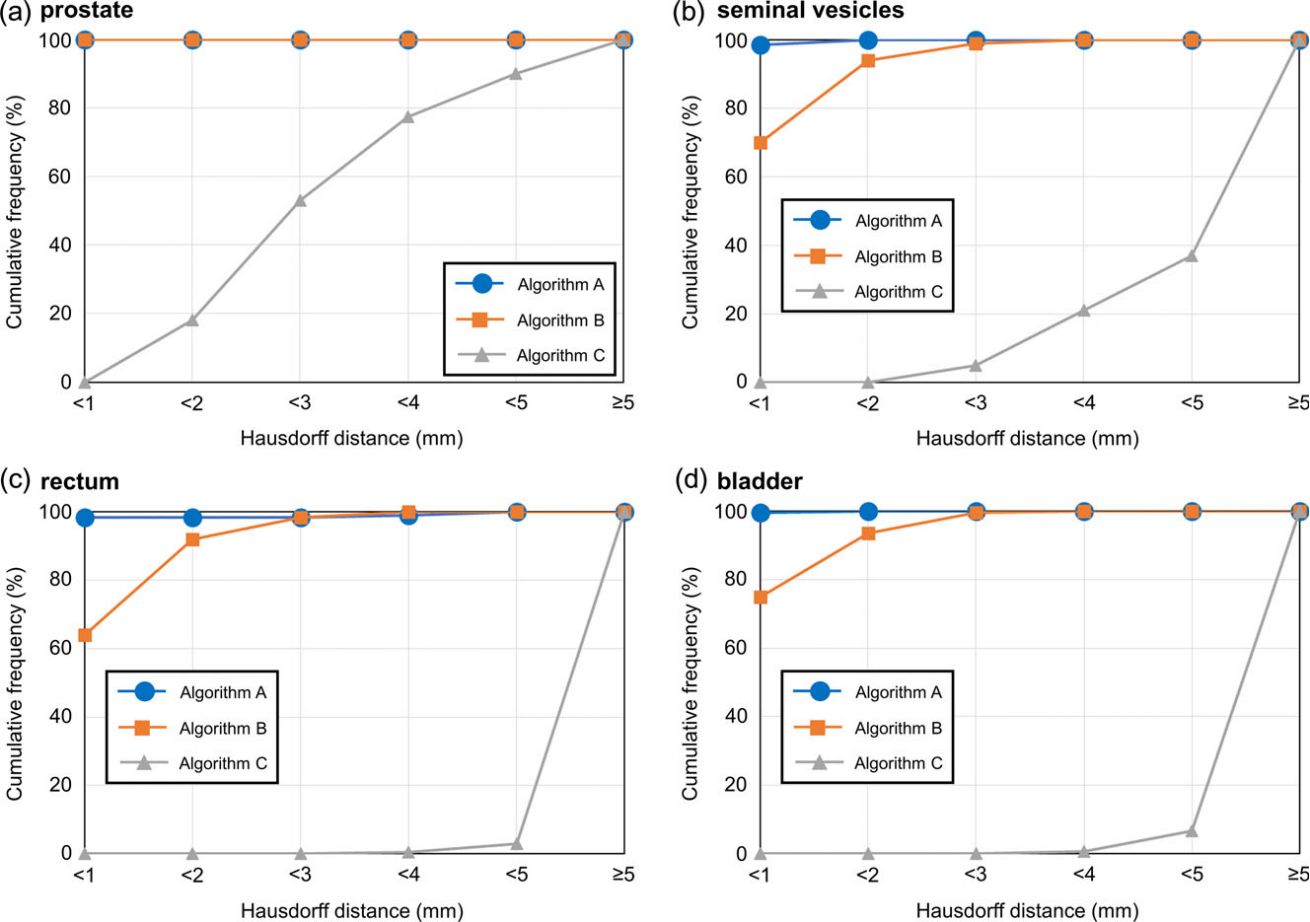
Cumulative histograms as a function of Hausdorff distance for the (a) prostate, (b) seminal vesicles, (c) rectum and (d) bladder. Algorithm A = 3D model-based global-to-local registration (Laplacian-based registration was used as a local registration); Algorithm B = 3D model-based global-to-local registration (finite element method-based registration was used as a local registration); Algorithm C = 3D model-based global registration.

Figure 2 illustrates the cumulative histograms by organ. In the prostate, Algorithms A and B yielded Hausdorff distances of <1 mm; the proportion of Hausdorff distances <2 mm was 18% using Algorithm C. For the SVs, the proportion of Hausdorff distances <2 mm was 100% and 94% with Algorithms A and B, respectively, and for the rectum the values were 98.5% and 92%. A similar trend was observed for the bladder. The proportion of Hausdorff distances <2 mm was 0% for the SVs, rectum and bladder using Algorithm C.

Table 3 summarizes the median Hausdorff distance and the computation time required to complete registration. The 75th percentile Hausdorff distance was <1 mm with Algorithm A. However, the value was >1 mm with Algorithm B, except for the prostate. The Hausdorff distance was 3.9 mm for the prostate and >7.8 mm for the other organs using Algorithm C. The median computation time required to complete registration was <101, 30 and 16 s for Algorithms A, B and C, respectively.

**Table 3.** Median Hausdorff distance and computation time required to complete registration

	Algorithm A	Algorithm B	Algorithm C	*P*-value
Hausdorff distance				
Prostate (mm)	0.24 (0.20–0.30)	0.26 (0.23–0.33)	2.85 (2.22–3.92)	<0.05
Seminal vesicles (mm)	0.39 (0.30–0.52)	0.72 (0.47–1.07)	5.87 (4.22–7.80)	<0.05
Rectum (mm)	0.52 (0.42–0.62)	0.81 (0.62–1.30)	10.89 (8.28–13.68)	<0.05
Bladder (mm)	0.41 (0.33–0.52)	0.59 (0.43–1.00)	8.55 (6.70–10.56)	<0.05
Computation time				
Prostate (s)	53.77 (32.40–74.10)	13.41 (10.98–15.89)	3.82 (2.79–5.19)	<0.05
Seminal vesicles (s)	100.85 (72.86–137.64)	29.75 (21.90–45.24)	15.39 (6.98–32.73)	<0.05
Rectum (s)	44.10 (29.47–62.93)	8.75 (7.32–10.05)	3.83 (2.44–5.46)	<0.05
Bladder (s)	70.62 (50.02–105.01)	17.00 (13.67–21.09)	5.20 (3.46–8.93)	<0.05

The number of patients used for validation of registration accuracy was 200. The interquartile range is shown in parentheses.

Algorithm A = 3D model-based global-to-local registration (Laplacian-based registration was used as a local deformable registration); Algorithm B = 3D model-based global-to-local registration (finite element method-based registration was used as a local deformable registration); Algorithm C = 3D model-based global registration.

The ANOVA revealed significant differences among Algorithms A–C in Hausdorff distance and computation time. In addition, no significant difference was observed in the difference of Hausdorff distance between Algorithm A and B with Tukey’s multiple comparison test.

## DISCUSSION

To our knowledge, this is the first study to assess the accuracy of 3D model-based global-to-local registration via the FEM- and Laplacian-based registration in the prostate region. Compared with other relevant studies, the number of patients used for validation of registration accuracy herein was greater. The 3D model-based global-to-local registration, especially that implementing the Laplacian-based registration, provided high registration accuracy for all organs in the prostate region. This would dramatically improve the accuracy of contour-based registration in the field of radiotherapy.

When implementing 3D model-based global-to-local registration, important factors that should be considered include the number of patients used to construct the template models and the registration algorithms employed. No study thus far has investigated the impact of the number of patients included in template model construction on registration accuracy. We found that the Hausdorff distance became significantly smaller only in the bladder when using Algorithm B, after increasing the number of patients included during construction of the template models. As shown in Table 1, the bladder volume varied more widely among the patients relative to the other organs. For such an organ, the number of patients used to construct template models would be important for the FEM-based registration. Meanwhile, the 3D model-based global-to-local registration implementing the Laplacian-based registration yielded Hausdorff distances <1 mm for all organs (Table 2). Unlike the FEM-based registration, the Laplacian-based registration provided sufficient registration accuracy, even using the template models constructed from only 10 patients. The performance of the FEM-based registration was comparable to that of the Laplacian-based registration for small round organs, such as the prostate; however, in general, the Laplacian-based registration accurately represented organs with large curvature, such as SVs and the rectum, better than the FEM-based registration. Laplacian-based registration overcomes the instability problem in matching distant structures, is suitable for large-scale deformations and handles unstructured deformable mesh models with different vertex densities [14]. Laplacian surface optimization improves the triangle quality of a surface mesh and also preserves detail when improving the mesh quality through the selection of different weights [18].

After confirming the impact of the number of patients used to construct template models on the accuracy of 3D model-based global-to-local registration, we compared registration accuracy between 3D model-based global-to-local registration and 3D model-based global registration, using 200 prostate cancer patients. As with previous studies, template models were constructed using data from 20 patients [19–22]. As expected, the performance of the 3D model-based global registration was the poorest among the three different registrations, because local registration was not conducted. Budiarto *et al.* calculated the residual of the deformations of the prostate using a deformable registration, named TPS robust point matching (TPS-RPM), and showed that the mean residual errors were >1 mm [20]. In addition, the TPS-RPM did not consider preservation of the geometrical features of the surface. In our study, the median Hausdorff distances were <0.3 mm and <0.8 mm for the prostate using Algorithms A and B, respectively, while preserving the geometrical features of the surface.

**Fig. 3.**
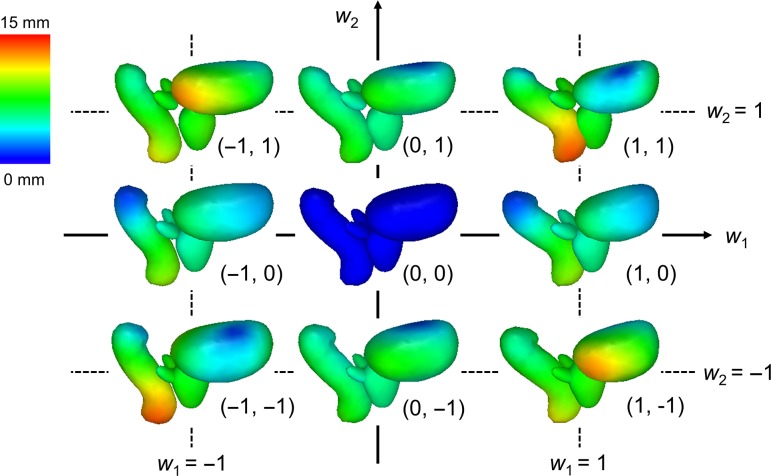
Samples of our statistical shape models derived from the resultant 200 prostate cancer patients using Algorithm A. The average model is located in the middle, and the colored parts illustrate displacement from that model. By varying weight parameters (*w*_1_ and *w*_2_), various shapes can be expressed.

The results of this study could be applied to the development of a new generation of statistical shape models (SSMs), which have been widely utilized in 3D modeling in recent years [23]. SSMs describe the shape of an object by applying principal component analysis to a set of landmarks, and are based on the assumption that each shape is a deformed version of a template model. In the field of radiotherapy, SSMs have been used as powerful tools to conduct segmentation [24, 25], estimate geometric uncertainties [19, 20] and calculate dose coverage [21, 22]. It is expected that detailed and robust SSMs will be generated by our 3D model-based global-to-local registration, according to the number of patients used to construct the template models and the registration accuracy (Fig. 3).

There were two notable limitations to this study. First, several radiation oncologists and medical physicists were involved in contouring; although they were well trained, interobserver variation should be included [26, 27]. However, any interobserver variation would have been averaged out as a large number of patients were included in this study. Secondly, we did not assess the applicability of our methods to other regions. As mentioned by Nakamura *et al.*, variations in organ shape in the upper abdominal region, such as of the stomach and duodenum, would be more complex than those of the rectum and bladder, even within the same patient [28]. In addition, livers are typically larger than bladders. Therefore, the applicability of our methods to other regions needs to be assessed.

In conclusion, we found that registration accuracy was not dependent on the number of patients used to construct template models for the Laplacian-based registration. Furthermore, the 3D model-based global-to-local registration implementing the Laplacian-based registration completed surface registration rapidly and provided better registration accuracy than that implementing the FEM-based registration for the prostate region.

## References

[1] National Cancer Institute at the National Institutes of Health Cancer Stat Facts: Prostate Cancer. Available from: https://seer.cancer.gov/statfacts/html/prost.html. Last accessed 20 September 2018.

[2] Zaorsky N, Palmer J, Hurwitz D et al What is the ideal radiotherapy dose to treat prostate cancer? A meta-analysis of biologically equivalent dose escalation. Radiother Oncol 2015;115:295–300. 26028229 10.1016/j.radonc.2015.05.011

[3] Reddy M, Nori D, Sartin W et al Influence of volumes of prostate, rectum, and bladder on treatment planning CT on interfraction prostate shifts during ultrasound image-guided IMRT. Med Phys 2009;36:5604–11. 20095273 10.1118/1.3260840 PMC13387077

[4] Tomita T, Nakamura M, Hirose Y et al Multivariate analysis of factors predicting prostate dose in intensity-modulated radiotherapy. Med Dosim 2014;39:360–5. 25155214 10.1016/j.meddos.2014.06.004

[5] Gianluca I, Roberto M, Elisabetta P et al Interfraction prostate displacement during image-guided radiotherapy using intraprostatic fiducial markers and a cone-beam computed tomography system: a volumetric off-line analysis in relation to the variations of rectal and bladder volumes. J Cancer Res Ther 2019 15(Suppl.):S69–75. 30900624 10.4103/jcrt.JCRT_463_17

[6] Brock K, Mutic S, McNutt T et al Use of image registration and fusion algorithms and techniques in radiotherapy: report of the AAPM Radiation Therapy Committee Task Group No. 132. Med Phys 2017;44:e43–76. 28376237 10.1002/mp.12256

[7] Vásquez Osorio E, Hoogeman M, Bondar L et al A novel flexible framework with automatic feature correspondence optimization for nonrigid registration in radiotherapy. Med Phys 2009;36:2848–59. 19673184 10.1118/1.3134242

[8] Jud C, Shandkühler R, Möri N et al Directional averages for motion segmentation in discontinuity preserving image registration. Medical Image Computing and Computer Assisted Intervention (MICCAI) 2017. LNCS 2017;10433:249–56.

[9] Krüger J, Ehrhardt J, Handels H Statistical appearance models based on probabilistic correspondences. Med Image Anal 2017;37:146–59. 28219833 10.1016/j.media.2017.02.004

[10] Nakao M, Minato K Physics-based interactive volume manipulation for sharing surgical process. IEEE Trans Inf Technol Biomed 2010;14:809–16. 20371420 10.1109/TITB.2010.2043460

[11] Suwelack S, Rohl S, Bodenstedt S et al Physics-based shape matching for intraoperative image guidance. Med Phys 2014;41:111901-1-12. 10.1118/1.4896021 25370634

[12] Saito A, Nakao M, Uranishi Y, et al Deformation estimation of elastic bodies using multiple silhouette images for endoscopic image augmentation. IEEE International Symposium on Mixed and Augmented Reality (ISMAR). 2015;170–1.

[13] Kim J, Valdes-Hernandez Mdel C, Royle N et al Hippocampal shape modeling based on a progressive template surface deformation and its verification. IEEE Trans Med Imaging 2015;34:1242–61. 25532173 10.1109/TMI.2014.2382581

[14] Drèan G, Acosta O, Lafond C et al Interindividual registration and dose mapping for voxelwise population analysis of rectal toxicity in prostate cancer radiotherapy. Med Phys 2016;43:2721–30. 27277019 10.1118/1.4948501

[15] Norihisa Y, Mizowaki T, Takayama K et al Detailed dosimetric evaluation of intensity-modulated radiation therapy plans created for stage C prostate cancer based on a planning protocol. Int J Clin Oncol 2012;17:505–11. 21960356 10.1007/s10147-011-0324-1

[16] Kim H, Park S, Lo S et al Bidirectional local distance measure for comparing segmentations. Med Phys 2012;39:6779–90. 23127072 10.1118/1.4754802 PMC4108711

[17] Huttenlocher D, Klanderman G, Rucklidge W Comparing images using the Hausdorff distance. IEEE Trans Pattern Anal Mach Intell 1993;15:850–63.

[18] Sorkine O, Cohen D, Lipman Y et al Laplacian surface editing. Proceedings of the 2004 Eurographics/ACM SIGGRAPH symposium on Geometry processing. 2004;175–84.

[19] Söhn M, Weinmann M, Alber M Modelling individual geometric variation based on dominant eigenmodes of organ deformation: implementation and evaluation. Phys Med Biol 2005;50:5893–908. 16333162 10.1088/0031-9155/50/24/009

[20] Budiarto E, Keijzer M, Storchi R et al A population-based model to describe geometrical uncertainties in radiotherapy: applied to prostate cases. Phys Med Biol 2011;56:1045–61. 21258137 10.1088/0031-9155/56/4/011

[21] Xu H, Vile D, Sharma M et al Coverage-based treatment planning to accommodate deformable organ variations in prostate cancer treatment. Med Phys 2014;41:101705-1-14. 10.1118/1.4894701 PMC4290467 25281944

[22] Tilly D, van de Schoot A, Grusell E et al Dose coverage calculation using a statistical shape model—applied to cervical cancer radiotherapy. Phys Med Biol 2017;62:4140–59. 28266348 10.1088/1361-6560/aa64ef

[23] Heimann T, Meinzer H Statistical shape models for 3D medical image segmentation: a review. Med Image Anal 2009;13:543–63. 19525140 10.1016/j.media.2009.05.004

[24] Pekar V, McNutt T, Kaus M Automated model-based organ delineation for radiotherapy planning in prostatic region. Int J Radiat Oncol Biol Phys 2004;60:973–80. 15465216 10.1016/j.ijrobp.2004.06.004

[25] Zhou J, Kim S, Jabbour S et al A 3D global-to-local deformable mesh model based registration and anatomy-constrained segmentation method for image guided prostate radiotherapy. Med Phys 2010;37:1298–308. 20384267 10.1118/1.3298374

[26] Fiorino C, Reni M, Bolognesi A et al Intra- and inter-observer variability in contouring prostate and seminal vesicles: implications for conformal treatment planning. Radiother Oncol 1998;47:285–92. 9681892 10.1016/s0167-8140(98)00021-8

[27] Choi H, Kim Y, Lee S et al Inter- and intra-observer variability in contouring of the prostate gland on planning computed tomography and cone beam computed tomography. Acta Oncol 2011;50:539–46. 21391773 10.3109/0284186X.2011.562916

[28] Nakamura A, Shibuya K, Nakamura M et al Interfractional dose variations in the stomach and the bowels during breath hold intensity-modulated radiotherapy for pancreatic cancer: implications for a dose-escalation strategy. Med Phys 2013;40:021701-1-9. 10.1118/1.4773033 23387724

